# Siglec-15 Promotes Tumor Progression in Osteosarcoma *via* DUSP1/MAPK Pathway

**DOI:** 10.3389/fonc.2021.710689

**Published:** 2021-07-16

**Authors:** Meng-ke Fan, Guo-chuan Zhang, Wei Chen, Li-li Qi, Ming-fang Xie, Yue-yao Zhang, Ling Wang, Qi Zhang

**Affiliations:** ^1^ Department of Orthopedic Research Center, The Third Hospital of Hebei Medical University, Shijiazhuang, China; ^2^ Department of Orthopedic Oncology, The Third Hospital of Hebei Medical University, Shijiazhuang, China; ^3^ Department of Orthopaedic Surgery, The Third Hospital of Hebei Medical University, Shijiazhuang, China; ^4^ Department of Pathogenic Biology, Hebei Medical University, Shijiazhuang, China

**Keywords:** osteosarcoma, Siglec-15, immune response, DUSP1, MAPK

## Abstract

Recurrence and metastasis are important features of osteosarcoma (OS) that cause its poor prognosis. Aberrant expression of Sialic acid-binding immunoglobulin-like lectin 15 (Siglec-15) has been reported in various kinds of cancers. However, the expression and function of Siglec-15 in OS remain unclear. In cultured OS cells (143B cells and MNNG/HOS cells) and their xenograft mouse models, we found that downregulation of Siglec-15 could inhibit the proliferation, migration and invasion of by inducing epithelial-mesenchymal transition (EMT) *in vitro* and *in vivo*. Conversely, Siglec-15 overexpression promoted the growth, migration and invasion of OS cells in a significant manner. Then, we screened a number of differentially expressed genes (DEGs) between Siglec-15-knockdown group and control group by RNA-Seq assay. Among these DEGs, we found that dual-specificity phosphatase 1 (DUSP1/MKP1) was significantly downregulated after Siglec-15 silencing. We investigated the DUSP1 functions in influencing OS cells’ biology, and found that the proliferation, migration and invasion of OS cells were promoted by overexpressing DUSP1 and crucially, the proliferation, migration and invasion of Siglec-15-knockdown OS cells were rescued by overexpressing DUSP1. Mechanically, we further showed that DUSP1-mediated inhibition of p38/MAPK and JNK/MAPK expression was attenuated when Siglec-15 expression was inhibited, suggesting that Siglec-15 promotes the malignant progression of OS cells by suppressing DUSP1-mediated suppression of the MAPK pathway. Moreover, we showed that both Siglec-15 and DUSP1 were highly expressed in human OS tissues by immunohistochemistry. High Siglec-15 expression was associated with OS lung metastasis, and high DUSP1 expression was associated with the high Enneking stage. Kaplan–Meier analysis indicated that high expression of Siglec-15 could predict poor prognosis of OS patients. Altogether, these results showed that Siglec-15 expression promoted OS development and progression by activating DUSP1 and might be a novel target in OS treatment.

## Introduction

Osteosarcoma (OS), which mostly occurs in teenagers, is a common primary bone neoplasm (1). The OS incidence is around one to three cases annually per million individuals, accounting for 20% to 40% of all bone cancers. OS presents a bimodal age distribution pattern, with peaks of incidence occurring under the average of 18 years old and another elderly crowd over 60 years old, respectively (2). There have been many recent advancements in the treatment of OS and the 5 year survival could almost reach 70-80%. However, some patients still have little or no response to recent treatment (2, 3). Importantly, 30%-40% of OS patients experience recurrence and metastasis in the short time after surgery, leading to a poor prognosis and a low survival rate to less than 20% of OS (2–6). Therefore, it is urgent to detect new methods for early diagnosis and treatment for OS.

Sialic acid-binding immunoglobulin-like lectins (Siglecs) are a family of transmembrane receptor-like glycan-recognition proteins. Based on sequence homology, Siglecs can be divided into two groups: the first group contains CD33-related Siglecs; the other group includes Siglec-1, Siglec-2, Siglec-4, and Siglec-15 (7, 8). These members transmit signals to the immunoreceptor tyrosine-based inhibitory motif/immunoreceptor tyrosine-based activation motif (ITIM/ITAM) by both activating and inhibiting receptors and participate in cell-to-cell recognition to regulate the immune response (7–10). Studies have shown that Siglec-15 is involved in bone growth, cancer, and infectious diseases (11–17). Activated macrophages expressing Siglec-15 can increase TGF-β secretion and inhibit the activity of CD4^+^ and CD8^+^ T cells by binding with the corresponding receptor to induce immune inhibition (18, 19). TGF-β is an important factor involved in the growth, spread and metastasis of cancer cells (20–22). The Siglec-15 gene is widely expressed in various cancers, such as lung cancer, liver cancer, kidney cancer and prostate cancer (19). However, the expression and functions of Siglec-15 in OS are unclear.

In this study, we explored the expression and function of Siglec-15 in OS cells *in vitro* and *in vivo* and found that the downregulation of Siglec-15 not only decreased proliferation but also decreased the migration and invasion of OS cells by inducing EMT and MMP-9 expression. Moreover, the downregulation of Siglec-15 decreased DUSP1 expression and promoted the accumulation of P38/MAPK and JNK/MAPK proteins. Overexpressing DUSP1 rescued the proliferation, migration and invasion abilities of shSiglec-15 cells. Our findings indicate that the Siglec-15/DUSP1/MAPK axis is a new molecular pathway involved in OS progression and may be an effective treatment strategy for OS development.

## Materials and Methods

### Cell Lines and Cell Culture

The human osteosarcoma cell lines 143B, KHOS, U20S, MNNG/HOS and SW1353 were obtained from CellCook (Guangzhou, China). The cell lines have been tested by MycoScan Mycoplasma Detection Kit (CellCook, China) and guaranteed without mycoplasma contamination. The cell lines were grown in DMEM medium (143B, KHOS and U20S), MEM medium(MNNG/HOS) or L15 medium (SW1353) supplemented with 10% fetal bovine serum (FBS) (Gibco, USA) and 1% penicillin/streptomycin (Thermofisher, USA), respectively. All of them were cultured at 37°C in a humidified 5% CO_2_ atmosphere. When cell confluence reaches 80% or more, the cells were digested by 0.25% Trypsin-EDT for further study. The completed medium was added to end the digestion when the cells shrink into a spherical shape under microscope (about 1-2 minutes). the cells was centrifuged at 1500rpm for about 5 minutes and then divide into 3-5 flasks for subculture.

### Antibodies and Reagents

Anti-Siglec-15 antibody (ab198684, rabbit, 1/100) and anti-slug antibody (ab27568, rabbit, 1/1000) were purchased from Abcam (Cambridge, UK); anti-N-cadherin antibody (22018-1-AP, rabbit, 1/5000), vimentin (10366-1-AP, rabbit, 1/5000), anti-MMP9 (10375-2-AP, rabbit, 1/1000), Ki-67 (27309-1-AP, rabbit, 1/5000). PCNA (10205-2-AP, rabbit, 1/1000), and GAPDH (60004-1-IG, mouse, 1/50000) were purchased from ProteinTech (Wuhan, China); and antibodies against p-DUSP1 (#2857, rabbit, 1/1000), JNK/MAPK(#9252, rabbit, 1/1000), p-JNK/MAPK (#4668, rabbit, 1/1000), P38/MAPK (#8690, rabbit, 1/1000), p-P38/MAPK (#4511, rabbit, 1/1000) were purchased from Cell Signaling Technology (Boston, USA). DUSP1 (bs-1851R, rabbit, 1/300) was purchased from Bioss (Beijing, China).

### RNA Interference and Plasmid Construction

Short hairpin RNA (shRNA) sequences were cloned into the lentiviral vector pGLV-H1-GFP-Puro by GeneChem (Shanghai, China) and cotransfected into OS cells with HiTransG P. The shRNA sequence for Siglec-15 was 5’-CTACGGAGAACTTGCTCAA-3’ (named shSiglec-15), and the matching empty vector (named shCtrl) was used as a negative control. To establish stable Siglec-15 knockdown, cells were seeded into a 6-well plate and then transfected with lentivirus (1×10^7^ TU/ml). After 48 h, puromycin (1 µg/ml) (Sigma, China) was used to select Siglec-15-knockdown OS cells for 10 days. Small interfering RNA (siRNA) against Siglec-15 was used for transient knockdown assays, which was synthesized by Ruibo Technology (Guangzhou, China). The siRNA sequences were as follows: siSiglec-15-A (5’-GCTCATTTGTGAGAACTAA-3’), siSiglec-15-B (5’-CTACGGAGAACTTGCTCAA-3’) and siSiglec-15-C (5’GGCCCAGGAGTCCAATTAT-3’). Cells were seeded into a 6-well plate and then transfected with 80 nmol/L siRNA with Hiperfect^®^ Transfection Reagent (QIAGEN, Germany). After 24 h, the cells were collected for the extraction of RNA or protein to test Siglec-15 expression or for other assays.

The pCMV-MCS-EGFP-SV40-Siglec-15 overexpression plasmid or pCMV-MCS-EGFP-SV40-DUSP1 overexpression plasmid were constructed by GenePharma (Shanghai, China) and transfected into OS cells with Lipofectamine^2000^ (Thermo Fisher, USA). After 48 h, G418 (200 μg/ml) (Cayman, USA) was used for selection of Siglec-15/DUSP1-overexpressing OS cells for 15 days.

### RNA Sequencing Assay

To identify the expressed differentiation of downstream genes between the Siglec-15-knockdown group and NC group, we performed RNA sequencing (RNA-Seq) analysis at LC-BIOTECHNOLOGIS (HANGZHOU, CO., LTD). At 36 h for OS cells transfected with siSiglec-15, total RNA was extracted using Trizol reagent (Invitrogen, CA, USA) following the manufacturer’s procedure. The total RNA quantity and purity were analysis of Bioanalyzer 2100 and RNA 1000 Nano LabChip Kit (Agilent, CA, USA) with RIN number >7.0. Poly(A) RNA is purified from total RNA(5μg) using poly-T oligo-attached magnetic beads using two rounds of purification. Following purification, the mRNA is fragmented into small pieces using divalent cations under elevated temperature. Then the cleaved RNA fragments were reverse-transcribed to create the final cDNA library in accordance with the protocol for the mRNA Seq sample preparation kit (Illumina, San Diego, USA), the average insert size for the paired-endlibraries was 300 bp ( ± 50 bp). And then we performed the paired-end sequencing on an IlluminaHiseq^4000^ at the (LC Sciences, USA) following the vendor’s recommended protocol. The transcript profiling data were subjected to Kyoto Encyclopedia of Genes and Genomes (KEGG) and Gene Ontology (GO) analysis.

### Real-Time PCR

Total RNA was extracted from cells using a mini kit (QIAGEN, Germany) and then RNA (50 mg/μL) was reversely transcribed into cDNA using the PrimeScript RT Reagent Kit (Takara, Japan). Realtime PCR was performed by using a 7500 real-time PCR system (Thermo Fisher, USA) with SYBR^®^ Green (Takara, Japan). We used the 2^-△△Ct^ method to determine the mRNA expression levels of genes. The primers were as follows: Siglec-15 (5’-CAGCCACCAACATCCATTTC-3’; 5’-CGCTCAAGCTAATGCGTGTA-3’), DUSP1 (5’-GCCACCATCTGCCTTGCTTACC-3’; 5’-ATGATGCTTCGCCTCTGCTTCAC-3’), and β-actin (5’-ctccatcctggcctcgctgt-3’; 5’-gctgtcaccttcaccgttcc-3’). Other primers are listed in **Figure S2**.

### Western Blotting

Transfected cells were lysed on ice for 35 min using RIPA buffer (Sigma, China) containing protease inhibitor (Roche, Switzerland) and phosphatase inhibitor (Thermo Fisher, USA) and then centrifuged for 15 min at 12,000 g at 4°C. The protein concentrations were analyzed using a BCA protein assay kit (Thermo Fisher, USA). Protein extracts (35 μg) were separated by 10%-12% SDS-PAGE (Sigma, China) and transferred to PVDF membranes (Millipore, USA). After blocking with 3%-5% nonfat milk, anti-Siglec-15, anti-N-cadherin, anti-Slug, anti-Vimentin, anti-MMP-9, anti-DUSP1, anti-p-DUSP1, anti-JUK/MAPK, anti-p-JNK/MAPK, anti-P38/MAPK, and anti-p-P38/MAPK antibodies were incubated with the PVDF membranes at 4°C overnight. The corresponding HRP-conjugated secondary antibodies were incubated with the PVDF membranes. Anti-GAPDH (ProteinTech, China) was used as an endogenous control. A chemiluminescence detection kit (Sigma, China) was used to detect the bands. Bio-Rad ChemiDoc MP all-round imaging system was used to detect the bands by A chemiluminescence detection kit (Sigma, China). The densitometry was performed by Image J software (Bethesda, USA).

### Cell Proliferation and Colony Formation Assays

Cell proliferation was evaluated by MTS (Promega, USA) assay. Transfected cells were seeded into a 96-well plate at 1.5×10^3^ cells per well (100 μL) and were then examined at different timepoint 24 h, 48 h, 72 h, 96 h and 120 h. MTS was added at a dilution of 1:4 to each well, and the cells were incubated at 37°C for 1 h. The absorbance value was detected at 492 nm by a microplate reader (PerkinElmer, USA). Cell colony numbers were evaluated by colony formation assays. Transfected cells were seeded into a 6-well plate at 1×10^3^ cells per well (2 mL) and cultured in DMEM or MEM for one week. Cells were fixed with methanol for 10 min and stained with 0.1% crystal violet (Sigma, China) for 20 min. The colonies (> 50) were counted by inverted light microscope (Ishikawa, Japan).

### Transwell Cell Migration and Invasion Assays

Cell migration and invasion assays were evaluated in 24-well Boyden chambers (Corning, USA). The wells were first coated with or without Matrigel (Corning, USA) and then incubated at 37°C overnight. Then, 600 µL of the corresponding medium containing 15% FBS was added to the lower chamber of the wells, and 3×10^4^ cells in serum-free medium were added to the upper chamber of the wells. After 24 h of 37°C incubation, the migrated cells were fixed in methanol for 15 min and stained with 0.1% crystal violet for 20 min. These cells were counted under an inverted light microscope (Ishikawa, Japan) in more than 3 random fields for each chamber.

### Wound Healing Assays

Cell migration was evaluated by wound healing assays. Transfected cells were seeded into a 6-well plate at 3.5×10^5^ cells per well and cultured in medium containing 10% FBS to 85%-90% confluence. Wounds were made by using a 10-µl pipette tip, and then the cells were cultured for 48 h using medium containing 2% FBS. Images were taken under an inverted light microscope at 0 h, 24 h and 48 h, then obtained the remaining area size of the scratches by Image J software (Bethesda, USA).

### Clinical Specimens and Immunohistochemistry

Thirty-six pairs of histologic sections of OS clinical samples were obtained from the Third Hospital of Hebei Medical University (Shijiazhuang, China). The enrolled patients were diagnosed to be OS according to clinical symptoms, imaging examination and pathological diagnosis, and all the patients had completed clinical parameters, including age, gender, location, size, Enneking stage, differentiation, recurrence, and lung metastasis. The excluded patients were with pregnancy, breastfeeding patients, other benign as well as malignant tumors, and severe cardiovascular and renal diseases. None of the patients received preoperative chemotherapy or radiotherapy. Informed consent was obtained from the patients before these samples were collected. The study was approved by the ethics committee of the Third Hospital of Hebei Medical University (NO. G2021-04-1).

According to the manufacturer’s instructions, IHC staining was performed using the UltraSensitiveTMSP (Rabbit) IHC Kit (ZSGB-Bio, China). The tissues were incubated with antibodies (anti-Siglec-15, anti-DUSP1, anti-N-cadherin, anti-Vimentin, anti-JUK/MAPK, anti-P38/MAPK, anti-Ki-67 and anti-PCNA) at 4°C overnight after the tissues were dewaxed and rehydrated. After the corresponding secondary antibodies were added to the sections for 15 min at 37°C, DAB (ZSGB-Bio, China) chromogen staining, hematoxylin counterstaining and dehydration were performed. The results of preliminary experiments were used to be positive control and the PBS instead of primary antibody were used to be negative control. The results were analyzed by two pathologists respectively. Through the analysis of ImageJ software (National Institutes of Health, USA), the percentage of positively stained cells was scored as 1 for ≤ 33%, 2 for 33% - 66%, 3 for ≥66%, and Siglec-15 or DUSP1 staining was scored 1 for absent/weak, 2 for moderate and 3 for strong. The two scores were multiplied to generate a total immune score, which as classified as a low score (0-3) or a high score (4-9) for every pathological section.

### Nude Mouse Tumor Model

Male or female BALB/c nude mice (6-8 weeks old), which were bred under specific-pathogen-free conditions, were purchased from Charles River (Beijing, China). The nude mice were randomly divided into the stable Siglec-15-knockdown (shSiglec-15) group and the matching empty vector (shCtrl) group (7 mice/group). OS cells (4×10^6^) with stable Siglec-15 knockdown or the matching empty vector cells were subcutaneously injected into the left or right axilla of nude mice. Tumor growth was measured every 3 days and was calculated by the formula V=0.5×(width)^2^×length (23). After 24 days, the mice were sacrificed, and the tumors were removed and weighed. IHC was performed to measure the expression of Siglec-15, DUSP1, Vimentin, P38/MAPK, PCNA and Ki-67 in tumor tissues. Animal experiments were performed according to protocols approved by the Third Hospital of Hebei Medical University Animal Care and Use Committee.

### Statistical Analysis

All assays were repeated three times. Graphs were created with GraphPad Prism software 8.0 (San Diego, CA, USA), and all results were analyzed with SPSS software version 21.0 (Chicago, IL, USA). T-tests was used to compare the means between 2 groups, and one-way analysis of variance was used to compare 3 or more groups. The chi-square test was used to determine the associations between Siglec-15/DUSP1 expression and clinical parameters. The overall survival rates were compared using Kaplan-Meier analysis. Correlations between two genes were analyzed using Pearson’s correlation analysis. *P* < 0.05 was considered statistically significance.

## Results

### Siglec-15 Promotes OS Cell Proliferation and Colony Formation *In Vitro*

**Figure 1:**
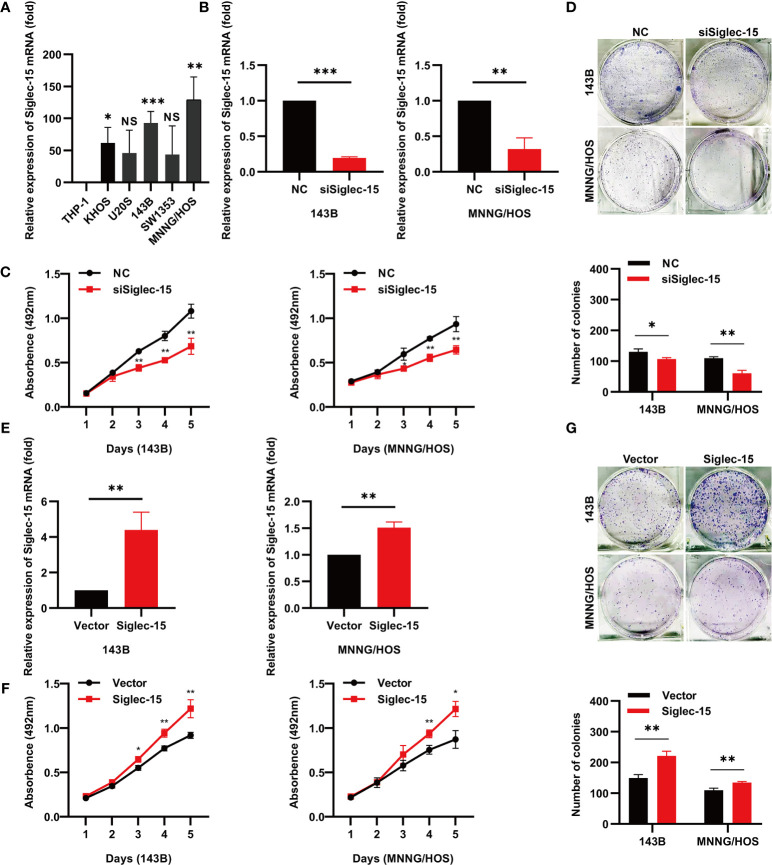
Siglec-15 promotes OS cell proliferation and colony formation *in vitro*. **(A)** Siglec-15 expression in OS cell lines was assessed by RT-PCR. **(B, C)** RT-PCR showed Siglec-15 knockdown in OS cells. **(D)** Cell proliferation assay and colony formation assay were performed on siSiglec-15 group and NC group. **(E)** RT-PCR showed Siglec-15 overexpression in OS cells. **(F)** Cell proliferation assay were performed on Siglec-15 group and Vector group. **(G)** Cell colony formation assay were performed on Siglec-15 group and Vector group. *P < 0.05, **P < 0.01, ***P < 0.001. NS, no significance.

First, we examined Siglec-15 expression in OS cell lines (**Figure 1A**) and found that Siglec-15 expression was higher in 143B cells and MNNG/HOS cells than in other cell lines. To further evaluate the biological function of Siglec-15 in OS cells, we used three different siRNAs to knockdown Siglec-15 expression in two OS cell lines, 143B and MNNG/HOS (**Figures S1A, B** and **1B**). Subsequently, we performed cell proliferation and colony formation assays and found that cell proliferation was significantly decreased in the Siglec-15-targeted siRNA sequence group (siSiglec-15) compared to the negative control group (NC) (**Figures 1C, D**). We then overexpressed Siglec-15 in both 143B cells and MNNG/HOS cells by transfection of the Siglec-15 plasmid (Siglec-15) and found that Siglec-15 overexpression promoted cell proliferation and colony formation compared with the matching empty vector (Vector) (**Figures 1E–G**).

### Siglec-15 Promotes OS Cell Migration and Invasion *In Vitro*

**Figure 2:**
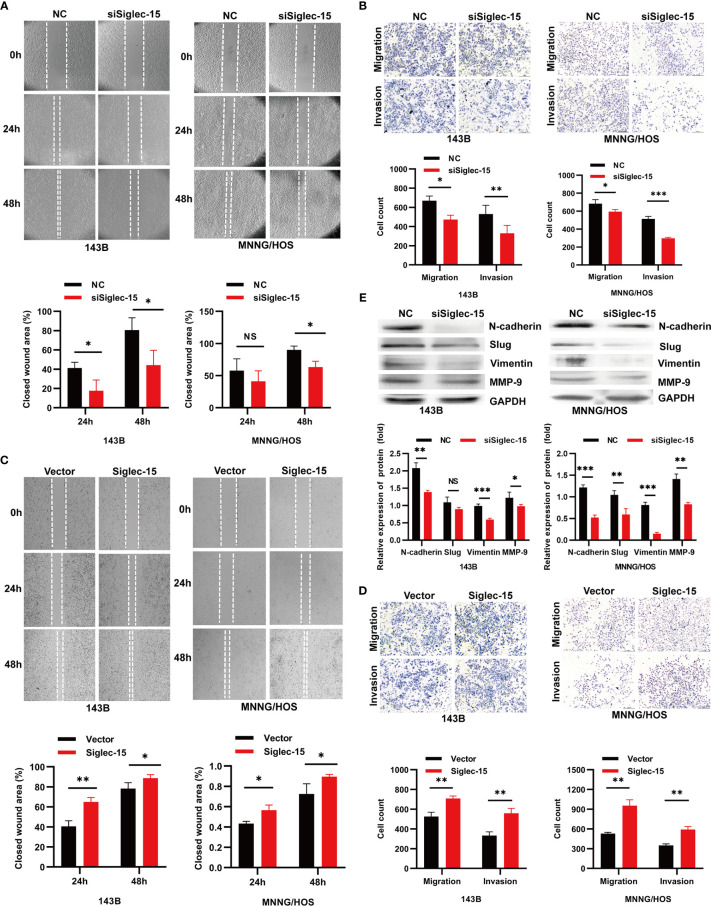
Siglec-15 promotes OS cell migration and invasion *in vitro*. **(A, B)** Wound healing assay and Transwell invasion assay were performed on siSiglec-15 group and NC group. **(C, D)** Wound healing and Transwell invasion assays were performed on Siglec-15 group and Vector group. **(E)** Western blotting analysis showed the levels of, N-cadherin, Slug, Vimentin, and MMP-9 in siSiglec-15 group and NC group. *P < 0.05, **P < 0.01, ***P < 0.001, NS, no significance. Scale bars, 400 µm and 200 µm.

We next investigated whether silencing or overexpressing Siglec-15 can affect the migration and invasion of OS cells *in vitro*. The migration and invasion abilities of OS cells were lower in the siSiglec-15 group than in the NC group according to Transwell assays and wound healing assays (**Figures 2A, B**), whereas the migration and invasion abilities of OS cells were increased in the Siglec-15 group compared with the NC group (**Figures 2C, D**). EMT related molecules and MMP family members have been widely used to predict cancer development and metastasis. Therefore, we also detected the expression of EMT markers, such as N-cadherin, vimentin, Slug and MMP-2/9 in OS cells to further evaluate the metastatic potential of OS cells. Western blotting analysis showed that the Siglec-15 knockdown group had decreased levels of N-cadherin, vimentin, Slug and MMP-9 compared with the NC group (**Figure 2E**).

### DUSP1 Is the Potential Target Regulated by Siglec-15 In OS Cells

**Figure 3:**
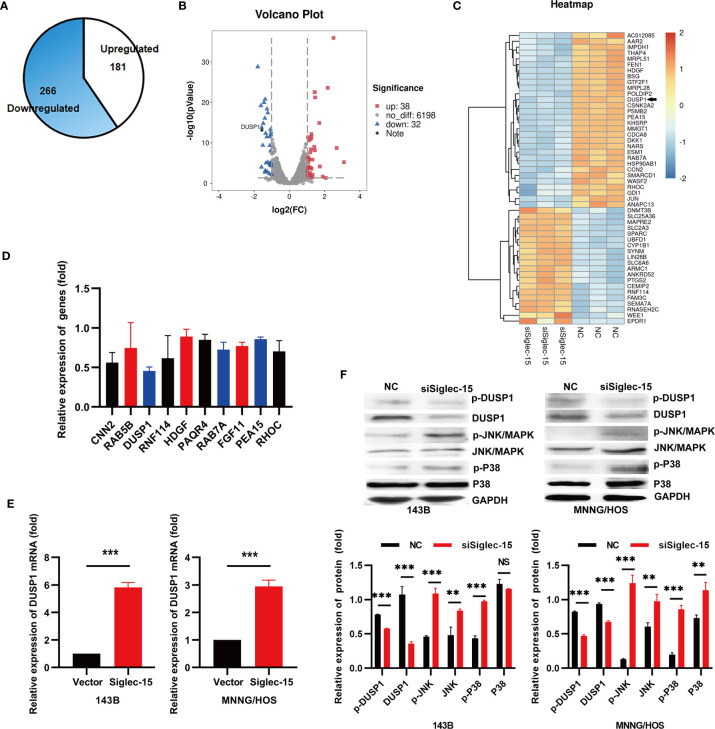
Siglec-15 promotes DUSP1 expression in OS cells. **(A)** Identification of genes regulated by Siglec-15 in OS cells using RNA-Seq assay. **(B, C)** A heat map and a volcano plot were constructed from the DEGs between siSiglec-15 group and NC group. **(D)** The top 10 significantly downregulated genes were analyzed by RT-PCR. **(E)** DUSP1 gene expression in siSiglec-15 group and NC group. **(F)** Western blotting assay showed that compared with NC group, DUSP1 and p-DUSP1 expression deceased, but p-JNK/MAPK, JNK/MAPK, p-P38/MAPK and P38/MAPK activation increased in siSiglec-15 group. **P < 0.01, ***P < 0.001, NS, no significance.

To further clarify the underlying mechanism by which Siglec-15 promotes OS activity, we carried out an RNA-Seq assay of OS cells (143B cells) in the siSiglec-15 group and NC group. The results showed that 266 genes were significantly downregulated (fold change>2) and 181 genes were significantly upregulated (fold change>2) in the siSiglec-15 group compared with the NC group (**Figure 3A**). A heat map and volcano plot were constructed for the siSiglec-15 group and the NC group and showed that genes were differentially expressed between the two groups (**Figures 3B, C**). Moreover, GO analysis of the differentially expressed genes revealed the top 10 significantly enriched terms related to these genes (**Figure 3D**). We then used realtime PCR to select and confirm the most significant related gene DUSP1, which could be significantly regulated by Siglec-15 in OS cells (**Figure 3E**). **Figures S1C** and **3E** data showed the DUSP1 expression in siSiglec-15 OS cells and Siglec-15-overexpressing OS cells. DUSP1 plays an important role in the MAPK pathway, and here we found it as a downstream target for regulated by Siglec-15 in OS cells.

DUSP1 acts as a tumor suppressor by negatively regulating MAPK activity in different tumors. To investigate whether Siglec-15 is involved in the activation of DUSP1, we performed Western blotting to detect in 143B and MNNG/HOS cells transfected with siSiglec-15 and found that Siglec-15 knockdown decreased DUSP1 and p-DUSP1 expression, but promoted the activation of p-JNK/MAPK, JNK/MAPK, p-P38/MAPK and P38/MAPK (**Figure 3F**), indicating that Siglec-15 might regulate DUSP1-mediated MAPK pathway.

### DUSP1 Counteracts the Inhibition Effects of Siglec-15 on the Biology in OS Cells

**Figure 4:**
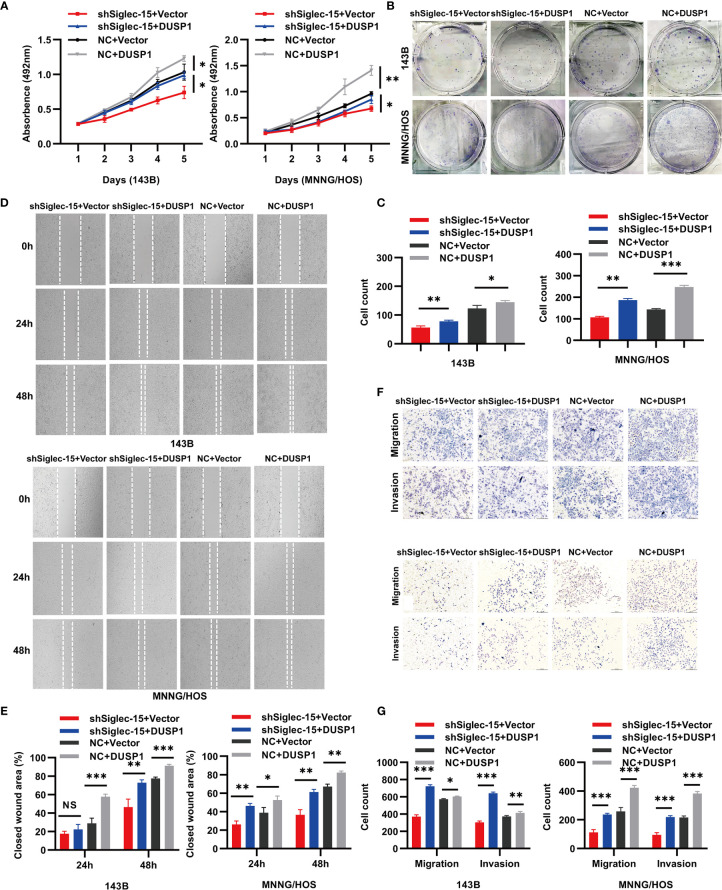
DUSP1 reverses the effects of shSiglec-15 on the proliferation, migration and invasion of OS cells. **(A–C)** NC+DUSP1 group increased the proliferation and clone formation of OS cells compared with NC+Vector group, and shSiglec-15+DUSP1 group also increased the proliferation and clone formation OS cells compared with shSiglec-15+Vector group. **(D–G)** NC+DUSP1 group promoted the migration and invasion of OS cells compared with NC+Vector group, and shSiglec-15+DUSP1 group rescued the migration and invasion of OS cells compared with shSiglec-15+Vector group. *P < 0.05, **P < 0.01, ***P < 0.001, NS, no significance. Scale bars, 400 µm and 200 µm.

To investigate the role of DUSP1 in resisting Siglec-15-promoted proliferation, migration and invasion, we constructed overexpression plasmid DUSP1 to transfect in 143B and MNNG/HOS cells with stable Siglec-15 downregulation. Results showed that overexpressed DUSP1 (NC+DUSP1) group increased cells proliferation and clone formation of 143B cells and MNNG/HOS cells compared with control (NC+Vector) group, and overexpressed DUSP1 in shSiglec-15 cells (shSiglec-15+DUSP1) also increased the proliferation and clone formation of 143B cells and MNNG/HOS cells compared with control (shSiglec-15+Vector) group (**Figures 4A–C**). In wound healing and Transwell assays, overexpressed DUSP1 (NC+DUSP1) group promoted the migration and invasion of 143B cells and MNNG/HOS cells compared with control (NC+Vector) group, and overexpressed DUSP1 in shSiglec-15 cells (shSiglec-15+DUSP1) rescued the migration and invasion of 143B cells and MNNG/HOS cells compared with control (shSiglec-15+Vector) group (**Figures 4D–G**).

### Siglec-15 Promotes OS Cell Growth by Activating EMT *In Vivo*

**Figure 5:**
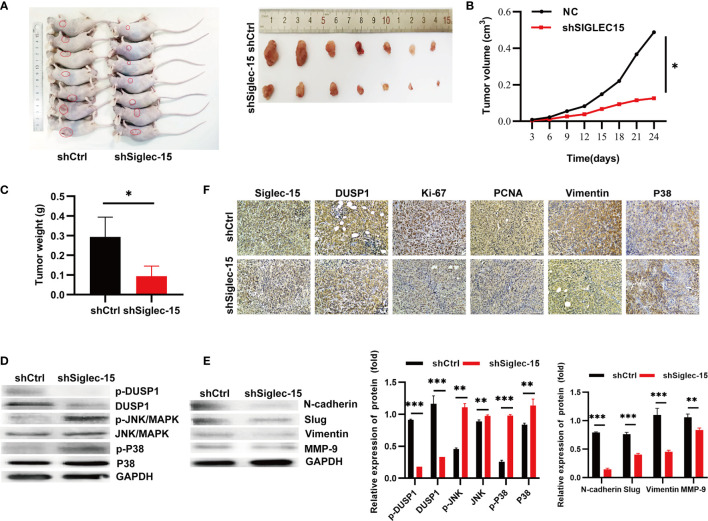
Siglec-15 promotes OS growth *in vivo* by inducing EMT. **(A)** The mice were sacrificed for tumor harvesting after 24 days and tumor image. **(B)** The tumor growth curves were plotted in shSiglec-15 group and shCtrl group. **(C)** Tumor weight was measured in shSiglec-15 group and shCtrl group. **(D)** Western blotting assay showed the expression of N-cadherin, Slug, Vimentin, and MMP-9 in shSiglec-15 group and shCtrl group. **(E)** Western blotting assay showed DUSP1, p-DUSP1, MAPK/JNK, p-MAPK/JNK, MAPK/P38, and p-MAPK/P38 in shSiglec-15 group and shCtrl group. **(F)** IHC staining for Siglec-15, DUSP1, Vimentin, P38/MAPK, PCNA and Ki-67 in in shSiglec-15 group and shCtrl group. *P < 0.05, **P < 0.01, ***P < 0.001.

To further explore whether Siglec-15 influences the proliferation and invasion of OS cells *in vivo*, we established the stable Siglec-15 silencing OS cell lines, and the transfection and knockdown efficiency were presented in **Figures S1D, E**. Next, we inoculated shSiglec-15 OS cells and shCtrl OS cells into nude mice, and the mice were sacrificed for tumor harvesting at 24 days (**Figure 5A**). The tumor sizes were significantly smaller in the shSiglec-15 group than in the NC group (**Figure 5B**). Tumor weights were also lower in the shSiglec-15 group than in the NC group (**Figure 5C**).

To illustrate whether the MAPK pathway, EMT, and MMP-9 participated in OS progression *in vivo*, we used Western blotting analysis and found that DUSP1 and p-DUSP1 decreased in tumors with Siglec-15 knockdown, whereas p-JNK/MAPK, JNK/MAPK, p-P38/MAPK and P38/MAPK increased in the shSiglec-15 group than in the NC group (**Figure 5D**). The expressions of EMT markers (N-cadherin, Slug, and vimentin) and MMP-9 decreased in shSiglec-15 group than that in NC group (**Figure 5E**). In addition, IHC analysis showed the levels of Siglec-15, DUSP1, Ki-67, PCNA, P38/MAPK and Vimentin were lower in shSiglec-15 group than that in shCtrl group (**Figure 5F**).

### Both Siglec-15 and DUSP1 Are Overexpressed in Human OS Specimens

**Figure 6:**
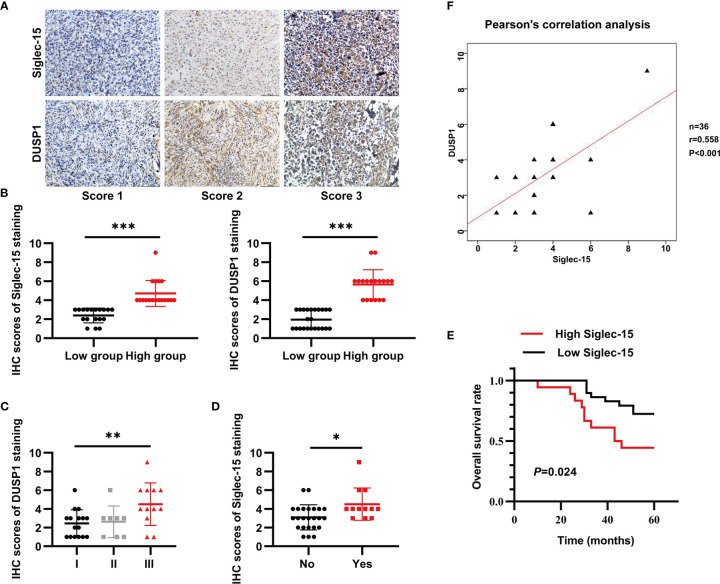
Both Siglec-15 and DUSP1 are expressed in OS specimens. **(A)** Representative image of Siglec-15 protein and DUSP1 protein in OS tissue at different stages. **(B)** The IHC scores of Siglec-15 and DUSP1 protein in high expression group and low expression group. **(C, D)** The IHC scores of DUSP1 for the Enneking stage and the IHC scores of Siglec-15 for lung metastasis. **(E)** Kaplan-Meier analysis between low Siglec-15 expression group and high Siglec-15 expression group. **(F)** The correlation between Siglec-15 expression and DUSP1 expression by the Pearson’s correlation analysis. *P < 0.05, **P < 0.01 and ***P < 0.001. Scale bars, 100 µm, in the lower right of photos.

To determine the protein levels of both Siglec-15 and DUSP1 in human OS tissues, we performed IHC staining and found that both Siglec-15 and DUSP1 were highly expressed in human OS tissues and mainly presented in cell membrane and cytoplasm of OS cells (**Figures 6A, B**). In human OS, Siglec-15 expression was positively associated with lung metastasis, and DUSP1 expression was positively associated with Enneking stage. However, no association was observed for these or other indexes (**Table 1**). The Siglec-15 staining scores were higher in patients with lung metastasis than in those without lung metastasis (**Figure 6C**), and the DUSP1 staining scores were higher in patients with Enneking stage III disease (**Figure 6D**). Kaplan-Meier analysis showed that the overall survival rate in high Siglec-15 group was lower than that in low Siglec-15 group (*P* = 0.024) (**Figure 6E**). The Pearson’s correlation analysis showed a positive correlation between the levels of Siglec-15 and DUSP1 in human OS (**Figure 6F**).

**Table 1:** Relationship between clinicopathologic parameters and Siglec-15 and DUSP1 expression in human osteosarcoma tissues.

Variables	Total patients	Siglec-15			*P* value	DUSP1		*P* value
		High		Low		High	Low
Age (mean)								
≤20	14	7		7	0.734	5	9	1.000
>20	22	9		13		9	13	
Gender								
Male	19	6		13	0.179	7	12	1.000
Female	17	10		7		7	10	
Tumor location								
Femur	26	13		13	0.198	11	15	0.874
Tibia	4	3		3		2	4	
Others	4	0		4		1	3	
Size (cm)								
≥5	28	15		13	0.053	10	18	0.683
<5	8	1		7		4	4	
Ennecking stage								
I	16	5		11	0.103	8	8	0.014
II	9	3		6		0	9	
III	11	8		3		6	5	
Differentiation								
High	10	6		4	0.441	5	10	0.909
moderate	11	5		6		5	6	
Low	15	5		10		4	6	
Recurrence								
Yes	6	3		3	1.000	4	2	0.181
No	30	13		17		10	20	
Lung metastasis								
Yes	25	8		17	0.034	8	17	0.273
No	38	20		18		12	26

## Discussion

Siglec members can recognize sialoglycans on immune cells or tumor cells to cause stimulatory or inhibitory effects (24). Siglec-15, first reported in 2001, is a new member of Siglecs and has been involved in many tumors’ development (19, 25, 26). In our studies, we first showed that the Siglec-15 is overexpressed in OS tissue and is mainly expressed in the cytoplasm and membrane of OS cells. High Siglec-15 expression was associated with tumor lung metastasis. In addition, there was a high correlation between high Siglec-15 expression and a low patient survival rate in OS patients. Therefore, we predict that Siglec-15 might be also involved in OS malignancy. In a series of *in vitro* experiments, we found that inhibiting Siglec-15 expression could suppress the proliferation and decrease the aggressiveness of OS cells. *In vivo*, we observed that the mice received Siglec-15 knockout OS cells exhibited reduced tumor growth and prolonged survival, which shared the similar results obtained in melanoma model from the study of Wang et al. (19). These results indicate that Siglec-15 plays an important role in promoting OS tumor growth. More evidence might be needed to support its role in the lung metastasis of OS *in vivo.*

Metastatic and recurrence are important to influence the prognosis of cancer patients, and these factors are related to tumor exacerbation, organ failure in metastatic site, and patients’ death. OS is highly prone to develop metastasis and recurrence, closely associated with a high rate of death (27–30). EMT is a crucial mechanism that enhances the transition of epithelial cells to mesenchymal cells to facilitate the metastasis of tumor cells (31, 32). Invasion, metastasis, immune evasion, and drug resistance would increase when tumor cells undergo EMT (33, 34). Studies revealed that EMT in OS could promoted tumor growth and metastasis (35–38). In our studies, we found that downregulation of Siglec-15 could not only reduce the expression of EMT-activating transcription factors (Slug), but also reduce the expression of mesenchymal markers (N-cadherin and Vimentin), suggesting that EMT as a critical process can participate in OS metastasis regulated by Siglec-15.

Matrix metalloproteinases are zinc (Zn^2+^) dependent endopeptidases which cause degradation of extracellular matrix proteins such as collagen, laminin, and fibronectin and further lead to the extracellular matrix remodeling (39). MMP-9, depending on the metal ion such as Zn2^+^and Ca2^+^, is upregulated during diverse pathologies including arthritis, diabetes, and cancer (40). Studies showed that MMP-9 promoted tumor invasion and metastasis by the degradation of gelatin and collagens (41–43). In our studies, we detected that MMP-9 was decreased in Siglec-15-knockdown group than that in control group *in vitro* and *in vivo*. Therefore, Siglec-15 mediated MMP-9 expression could be a potential mechanism to help OS cells migrate and invade, further associated with poor prognosis of OS patients.

The JNK/MAPK, P38/MAPK and ERK/MAPK proteins are downstream proteins of MAPK signaling pathway, involved in various cellular processes, such as proliferation, differentiation, migration and apoptosis (44–47). DUSP1 is recognized as a key role in inactivating different MAPK isoforms, which joined in cell proliferation, differentiation, transformation, stress responses, inflammation, cycle arrest, and apoptosis (48–55). Studies found that high DUSP1 has been observed in various human cancers, including colon (56), bladder (57), gastric (58), breast (59), and lung cancer (60). Here, we found that DUSP1 expression was regulated by Siglec-15 in OS cells, and overexpression of DUSP1 could counteract Siglec-15-caused malignant biological characteristics *via* MAPK pathway. Thus, our results suggest that DUSP1 might become a potential therapeutic target for OS. Recently, it has been reported that DUSP1 expression promoted tumor proliferation through miR-34a, and associated with poor prognosis of OS patients (61). Therefore, our next step could explore the upstream regulatory mechanism of the Siglec-15-DUSP1 axis in OS progression, which would further confirm our hypothesis and make Siglec-15 possible for future clinical application.

In order to testify the clinical relationship between the expression of Siglec-15 and DUSP1, IHC staining were used to detect these protein levels in OS patients. We found that both Siglec-15 protein and DUSP1 protein were highly expressed in human OS tissues. High Siglec-15 expression was associated with OS lung metastasis and high DUSP1 expression was associated with high Enneking stage. Kaplan–Meier analysis indicated that high expression of Siglec-15 was associated with poor prognosis of OS patients. Therefore, Siglec-15-DUSP1 axis might an important alternative mechanism to promote development and progression of human OS.

In conclusion, our study demonstrated that Siglec-15 was abnormally expressed in tissues and cells of OS. High Siglec-15 expression promoted the proliferation, migration and invasion of OS cells by DUSP1-inactivated MAPK pathway, suggesting that targeting Siglec-15 may be a novel therapeutic strategy for OS patients.

## Data Availability Statement

The datasets presented in this study can be found in online repositories. The names of the repository/repositories and accession number(s) can be found below: GSE175414.

## Ethics Statement

The studies involving human participants were reviewed and approved by third hospital of Hebei medical university. Written informed consent to participate in this study was provided by the participants’ legal guardian/next of kin. The animal study was reviewed and approved by third hospital of Hebei medical university.

## Author Contributions

M-kF conducted the *in vivo* experiment and WC and G-cZ collected clinical samples and patient information. L-lQ and M-fX performed the cell experiments *in vivo*. LW and Y-yZ contributed to the analyses of all the data and composed the manuscript. LW and QZ helped design the experiment, provided materials and revised the manuscript. All authors contributed to the article and approved the submitted version.

## Funding

This work was supported by several grants from National Natural Science Foundation of China (81772858) and Hebei Natural Science Foundation (H2019206058).

## Conflict of Interest

The authors declare that the research was conducted in the absence of any commercial or financial relationships that could be construed as a potential conflict of interest.

## Supplementary Material

Supplementary Figure 1
**(A)** Transfection efficiency of siRNA in 143B cells and MNNG/HOS cells. **(B)** The silencing efficiency of Siglec-15 siRNA by RT-PCR in 143B cells. **(C)** DUSP1 gene expression in siSiglec-15 group and NC group. **(D)** Transfection efficiency of Siglec-15 shRNA in 143B cells. **(E)** The knockdown efficiency of Siglec-15 shRNA by RT-PCR in 143B cells. ****P* < 0.001. Scale bars, 40 µm and 100 µm.

Supplementary Figure 2 The primer sequences for validating the most significant 10 genes based on RNA-Seq assay for real time PCR.
