# Supplementary figures and images for: Siglec-15 Promotes Tumor Progression in Osteosarcoma via DUSP1/MAPK Pathway
Source: Front Oncol. 2021 Jul 16;11:710689. doi: 10.3389/fonc.2021.710689 (PMC8322944; doi:10.3389/fonc.2021.710689)

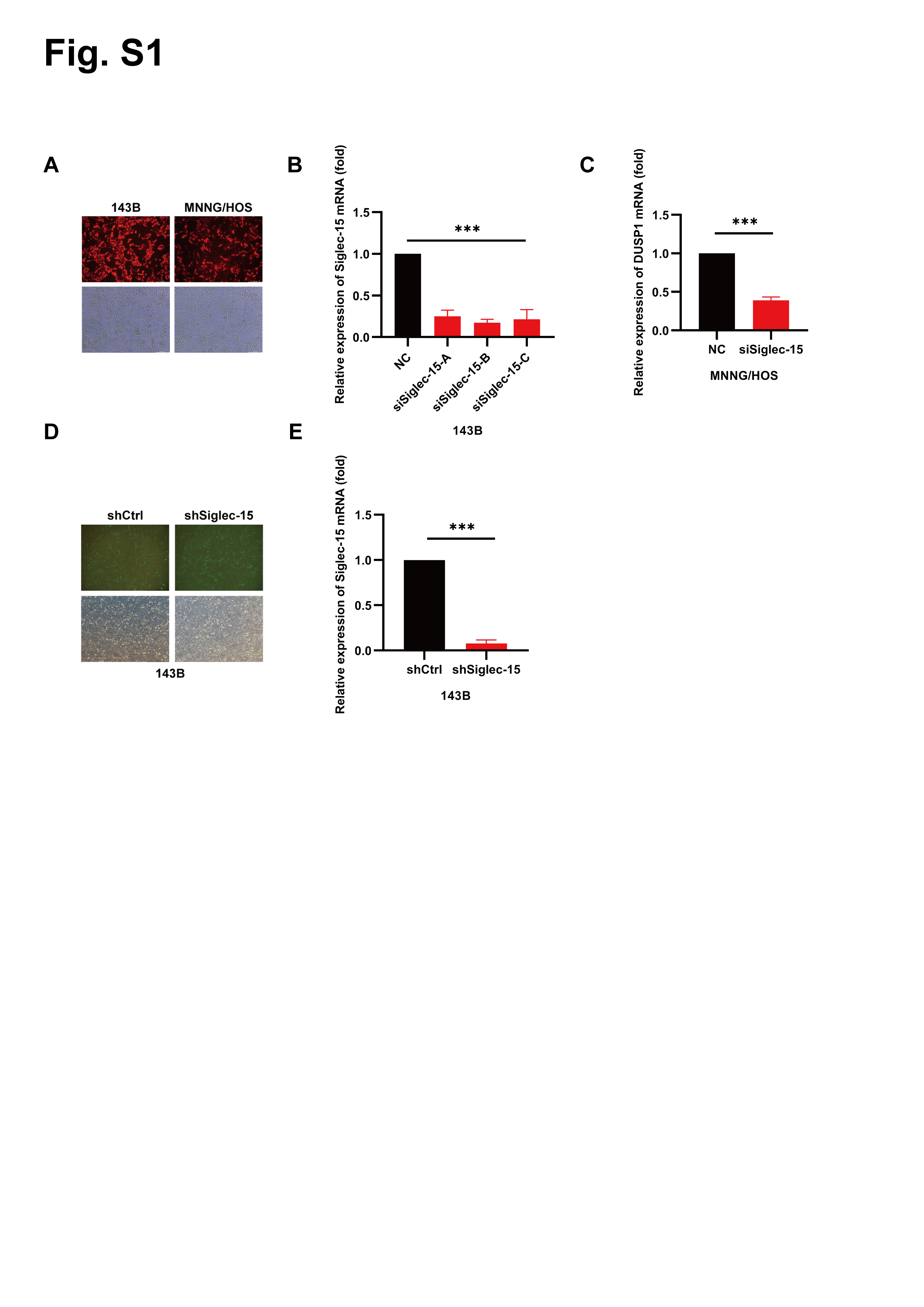

Supplement: Supplementary Figure 1 — (A) Transfection efficiency of siRNA in 143B cells and MNNG/HOS cells. (B) The silencing efficiency of Siglec-15 siRNA by RT-PCR in 143B cells. (C) DUSP1 gene expression in siSiglec-15 group and NC group. (D) Transfection efficiency of Siglec-15 shRNA in 143B cells. (E) The knockdown efficiency of Siglec-15 shRNA by RT-PCR in 143B cells. ***P < 0.001. Scale bars, 40 µm and 100 µm. [file Image_1.tif]

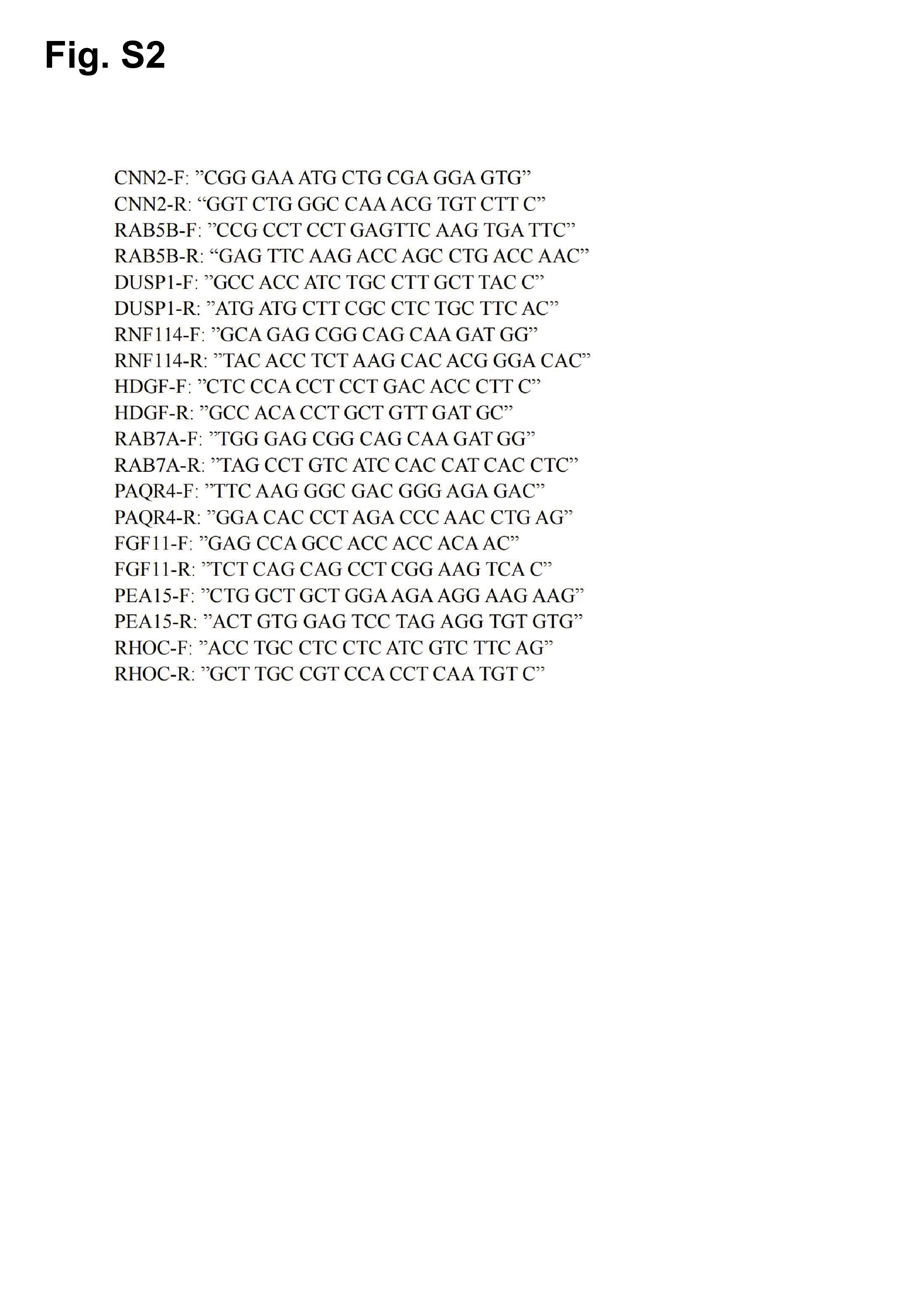

Supplement: Supplementary Figure 2 — The primer sequences for validating the most significant 10 genes based on RNA-Seq assay for real time PCR. [file Image_2.tif]
